# Discrepancies in the number of lines of arrested growth (LAG) in the tissues of the humerus and phalanx of sea turtles

**DOI:** 10.1007/s00114-025-01963-7

**Published:** 2025-01-23

**Authors:** Emre Sandık, Bektaş Sönmez, Şükran Yalçın Özdilek

**Affiliations:** 1 Sea Turtle Research and Application Center, Çanakkale Onsekiz Mart University, Çanakkale, Türkiye; 2 Suşehri Timur Karabal Vocational School, Sivas Cumhuriyet University, 58600 Suşehri Sivas, Türkiye; 3 Department of Biology, Faculty of Science, Çanakkale Onsekiz Mart University, Terzioğlu Campus, 17100 Çanakkale, Türkiye

**Keywords:** Age estimates, Lines of arrested growth (LAG), Humerus, Phalanx, *Caretta caretta*, *Chelonia mydas*

## Abstract

Information on the age of vertebrate species such as sea turtles is crucial for planning management and conservation actions. The age of sea turtles has been estimated by skeletochronological analysis using skeletal growth marks in different bones. This study focused on the consistency of the number of visible lines of arrested growth (LAG) observed from the humerus and phalanx bone used for age estimation in *Chelonia mydas* and *Caretta caretta*. We collected 67 humeri and phalanges of *C. mydas* (*n* = 47) and *C. caretta* (*n* = 20) from Samandağ beach, eastern Mediterranean in 2020–2022. LAG in the humerus and phalanx of the same individual were counted by two readers, and their consistency with each other was determined by percent agreement (PA), average percent error (APE), and coefficient of variation (CV). The significance of the difference between them was determined by the McNemar test. The mean number of visible LAG counted from the humerus is greater than the mean number of visible LAG counted from the phalanx, i.e., the humerus contains more growth marks than the phalanx. However, in individuals up to 15 LAG in *C. mydas* and 10 LAG in *C. caretta*, the mean number of visible LAG observed in both bone tissues is compatible. This was supported by the differences in the resorption rates calculated in both bones, indicating that the number of LAG lost due to resorption may also differ between these two bone types. It is recommended that the back calculation and/or correction factor applied for the humerus be avoided for the phalanx.

## Introduction

Effective conservation and management of threatened and endangered species such as sea turtles is important to measure demographic parameters such as somatic growth rates, time to maturity, and reproductive lifespan (Turner Tomaszewicz et al. 2022). Moreover, these demographic parameters are key parameters in models used to assess population dynamics (Avens et al. 2020; Turner Tomaszewicz et al. 2022). In addition to these, the determination of the age of individuals in sea turtle populations is also a highly useful tool for understanding their ecology (Avens et al. 2013; 2021). Because age determination allows for the acquisition of knowledge regarding the life history of species. With the assistance of age-based estimates, it is possible to examine parameters such as survival, mortality, and growth rates, as well as reproductive behavior (Baldi et al. 2023; Sanchez et al. 2023; Inoue and Ishihara 2024).

Numerous structures containing growth marks have been used to study age and growth in vertebrates. For example, fish otoliths, which continue to grow even after somatic growth has ceased in mature individuals (Lai et al. 2023), and dentin layers, which accumulate annually in continuously growing teeth in some mammals (Cebuhar et al. 2021), have been used to age. However, the most widely used method for estimating age and growth rates in vertebrates is skeletochronology, which has also been employed in many species of sea turtles (Guarino et al. 2020; Goshe et al. 2020; Şirin and Başkale 2021; Avens et al. 2020; 2021; Medetian and Miaud 2024). In skeletochronology, lines of arrested growth (LAG), which develop annually within long bones during unfavorable growth conditions, are used to estimate age (Castanet 1994). Furthermore, this technique has been used as a standard methodology for assessing individual growth rates in sea turtles (Avens et al. 2021; Sanchez et al. 2023; Inoue and Ishihara 2024; Medetian and Miaud 2024). The annual growth marks on the humerus bone have been validated for most sea turtles (Snover et al. 2011; Goshe et al. 2016). Due to the typical remodeling or resorption of periosteal bone, early growth marks are destroyed (Zug et al. 1986; Snover et al. 2007). It has therefore been proposed that a reliable method for estimating the number of resorbed growth marks in sea turtle limb bones needs to be established (Snover et al. 2007; Avens et al. 2013; 2015). Many authors have documented and attempted to compensate for the high levels of resorption observed in sea turtle limb bones using various analytical techniques such as the back-calculation method and correction factor equation (Snover et al. 2007; Avens et al. 2015; Turner Tomaszewicz et al. 2022; Medetian and Miaud 2024).

In addition to the humerus, the phalanx, femur, and scleral ossicle bones have also been utilized in age estimation studies of sea turtles (Zug et al. 1986; Avens et al. 2009; Guarino et al. 2020; Inoue and Ishihara 2024; Medetian and Miaud 2024). The question of which bone growth increments (layers or markings) can be most consistently observed was first investigated in sea turtles by Zug et al. (1986). The authors conducted histological examinations of various bones in the turtle, including the third peripheral bone from the carapace, the dentary, a centrum of a cervical vertebra, a penultimate phalanx of the forelimb, an ulna, and a humerus (Zug et al. 1986). The humerus was identified as the most suitable bone for further examination, as it exhibited the highest cortical/spongy bone ratio and the greatest number of growth markings (Zug et al. 1986).

Over the following years, several researchers have attempted to estimate the age of different bones. For instance, in studies of skeletochronology on the species *Dermochelys coriacea*, *Lepidochelys kempii*, and *Caretta caretta*, age estimation was conducted on scleral ossicles (Avens and Goshe 2007; Avens et al. 2009). However, due to the high resorption rate in older individuals, the age was underestimated in comparison to the humerus bone (Avens and Goshe 2007). Another example is phalanx skeletochronology. It has been documented that periosteal rings and growth marks can be observed in the phalanx bones and represent a reliable methodology for the estimation of age in *C. caretta* (Guarino et al. 2004; 2020). Nevertheless, it has been proposed that distinguishing growth marks from one another in phalanx bones at later ages may prove challenging (Guarino et al. 2004; 2020). This is due to the high resorption rate of phalangeal bones in older age groups (Guarino et al. 2004; 2020).

It can be observed that different authors employ disparate methodologies in the sea turtle age estimation techniques. Although various bones have been examined as candidate tissues for ageing studies, most sea turtle ageing studies focus on the humerus bone for all species except the few studies, where scleral ossicles and phalanx are used. However, there is a paucity of data regarding the consistency of age estimates derived from two distinct bone tissues (humerus and phalanx) of the same individual. Guarino et al. (2020) reported an age agreement of 85% for phalanx and humerus bones in 13 *C. caretta* individuals up to 15 years of age. However, it may not be applicable to all size/age classes due to the resorption rate at older ages (Guarino et al. 2004; 2020). Additionally, there is no comparable study on the age (or visible LAG count) consistency of two different bones in green turtles (*Chelonia mydas*). Therefore, this study aimed to test the consistency of the number of visible LAG between the humerus and phalanx of *C. mydas* and *C. caretta*.

## Materials and methods

### Sample collection

The humerus and phalanx bones of *C. caretta* and *C. mydas* for skeletochronological analysis were collected from Samandağ beach (36°07′K, 35°55′D) in the eastern Mediterranean in the 2020–2022 nesting seasons. This study was conducted under the permission and supervision of the Republic of Turkey Ministry of Agriculture and Forestry, General Directorate of Nature Conservation and National Parks (Protocol No. 21264211–288.04–6124877). All specimens were collected from dead stranded individuals, and no turtles were sacrificed to obtain specimens for this study. From each turtle, the right humerus and the median phalanx of the third phalange of the right forelimb were dissected, labeled in zip lock bags, and transported to the laboratory. Guarino et al. (2020) demonstrated that the estimation of age in the phalanx using the right fore flipper is a suitable method for this purpose. Consequently, in this study, the right fore flipper was selected to align with the existing literature. When the right limb was not available, the left limb was used. Dead stranded sea turtles were identified to species, and the curved carapace lengths (CCL) were measured using a flexible tape measure (to the nearest mm) (Sönmez 2019). Samples were decontaminated by boiling to remove soft tissues and dried outdoors for 2 weeks (Goshe et al. 2020; Guarino et al. 2020; Sandık et al. 2024).

### Skeletochronology

**Fig. 1:**
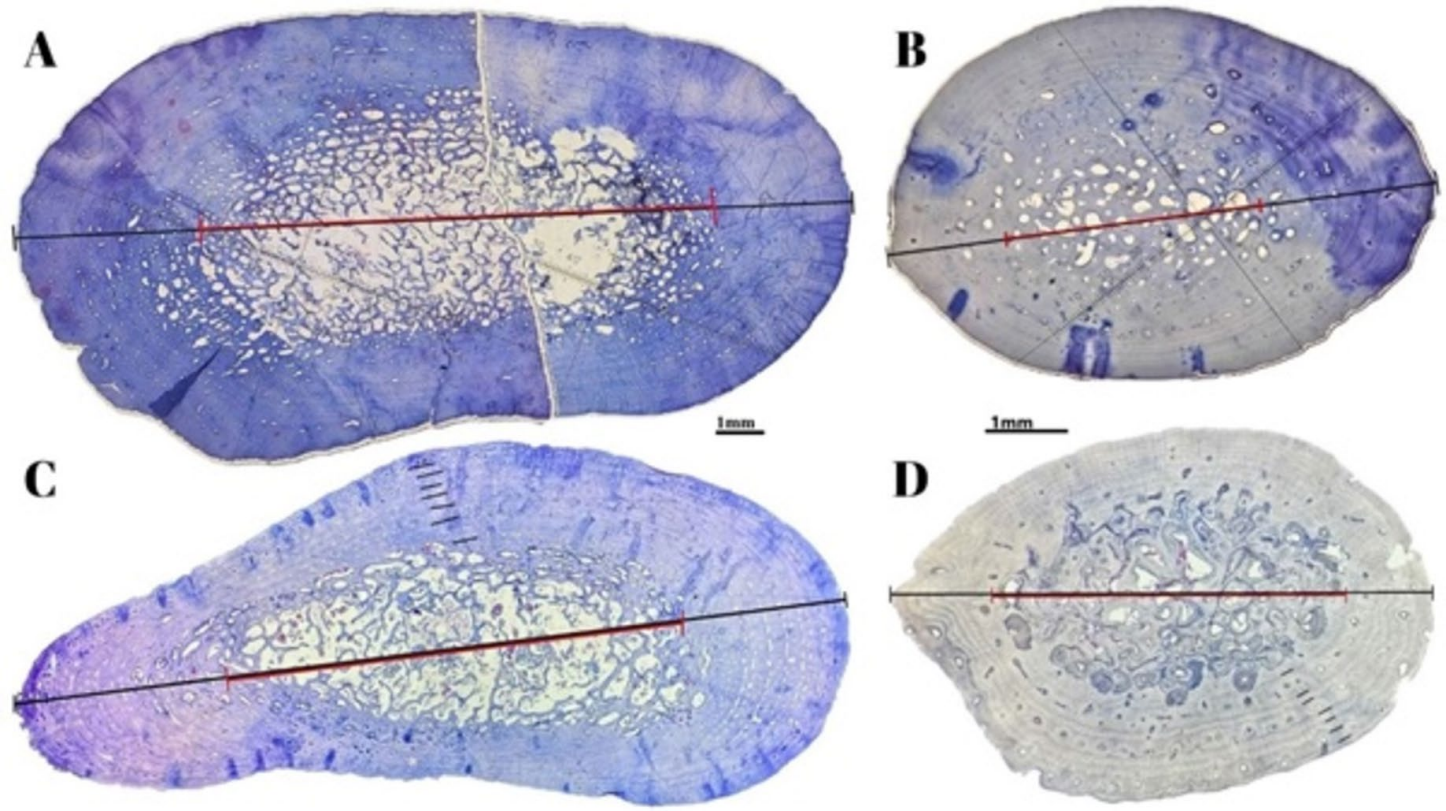
A comparative image of histological preparations of the humerus and phalanx from the same individual is provided alongside a general view of the horizontal diameter of the bone (total diameter) and the diameter of the resorption core. In the first case (**A**), the image is of the humerus of *C. mydas*, in the second (**B**), that of the phalanx of the same species. The next images (**C** and **D**) show the humerus and phalanx of *C. caretta*. The horizontal diameter of the bone is represented by a black line and the diameter of the resorption core by a red line (magnification 0.8 × for humeri, 2 × for phalanges)

Humerus and phalanx specimens were cut 3–6 mm wide from the narrowest part of the diaphysis with a hacksaw and placed in 10% neutral buffered formalin for fixation (Goshe et al. 2020). The fixed specimens were washed in distilled water and decalcified with a decalcifying solution (Bio-Optica, Biodec R, 05-03009Q) (Goshe et al. 2020; Guarino et al. 2020). Decalcified bones were soaked in water overnight to remove any residual decalcifying solution (Avens and Goshe 2007; Goshe et al. 2020) and then dehydrated through a series of graded ethanol series (Guarino et al. 2020). Specimens were then cleared in xylene, paraffin-embedded in Leica HistoCore Arcadia H, sectioned on a Leica RM 2145 RTS rotary microtome (7 µm), and subjected to routine hematoxylin (20 min) and eosin (dip-and-pull) staining (Zug et al. 1986). Microscopic examination was performed using an Olympus sz51 stereo binocular microscope (magnification 0.8 × for humeri, 2 × for phalanges). Comparative images of histological preparations of humerus and phalanx from the same individual are shown in Fig. 1. Counts of lines of arrested growth (LAG), which delineate the outer boundaries of skeletal growth marks (Lenz et al. 2016; Goshe et al. 2020; Guarino et al. 2020; Sandık et al. 2024), were performed by two observers.

The resorption rates in both bone tissues of both species were calculated as the ratio of the horizontal axis diameter of the bone (total diameter) to the diameter of the resorption core (Avens et al. 2013) (Fig. 1). Given the irregular structure of the resorbed area, the diameter of the innermost measurable LAG was referred to as the resorption core diameter (Avens et al. 2013).

### Data analysis

Humerus and phalanx specimens were read three times by each reader at different times regardless of size. The mean visible LAG count (*X*_j_) was calculated from the LAG count data using Eq. 1 (Baker and Timmons 1991), and was applied to the humerus and phalanx separately. In this formula, *X*_j_, mean LAG count; *n*, number of readings; *f*, number of samples; *X*_ij_, mean LAG count obtained at reading *i* for individual *j*.

1 $${\mathrm{x}}_{\mathrm{j}}=\frac{\sum_{\mathrm{i}}^{\mathrm{n}}\sum_{\mathrm{j}}^{\mathrm{f}}{\mathrm{x}}_{\mathrm{ij}}}{\mathrm{nf}}$$

The calculation of the percent agreement (PA) of visible LAG counts between readers and between bone tissues was based on the percentage of all samples in which all three of the three readings for each bony structure were the same (Kimura and Lyons 1991).

The average percentage error (APE) of visible LAG counts between readers and between bone tissues was calculated according to Eq. 2 (Beamish and Fournier 1981). In this formula; APEj, average percent error for individual *j*; *R*, number of readings; *X*_ij_, visible LAG count *i* reading of individual *j*; *X*_j_, mean visible LAG count for individual *j*.

2 $$AP{E}_{\dot{j}}=100\%\frac{1}{R}\sum_{i=1}^{R}\frac{\left|{X}_{i\dot{J}}-{X}_{j}\right|}{{X}_{j}}$$

The coefficient of variation (CV), which provides a statistical test of the repeatability of ageing between readers and bone tissues, was calculated according to Eq. 3 (Chang 1982). In this formula, CVj, coefficient of variation for individual *j*; *R*, number of readings; *X*_ij_, visible LAG count read of individual *j*; *X*_j_, mean visible LAG count for individual j.

3 $$C{V}_{j}=100\%\frac{\sqrt{\sum_{i=1}^{R}\frac{{\left({X}_{ij}-{X}_{j}\right)}^{2}}{R-1}}}{Xj}$$

An independent sample *t*-test was conducted to assess the discrepancy between the mean number of visible LAG and resorption rates in two distinct bones. Additionally, the agreement between the visible LAG counted in two different bone tissues was tested with the McNemar test (Pembury Smith and Ruxton 2020). The differences between the two different bone tissue readings were also shown in the Bland–Altman plot (Ogle 2017). However, the structure exhibiting the highest PA, the lowest APE, and the lowest CV was deemed the most reliable bony structure for the number of visible LAG counted for the determination of the species. Statistical analyses and modeling were conducted in the R programming, and the FSA (Ogle et al. 2023), readxl, and ggplot2 (Wickham 2011; Wickham et al. 2019) libraries were employed (R Core Team 2023).

## Results

**Fig. 2:**
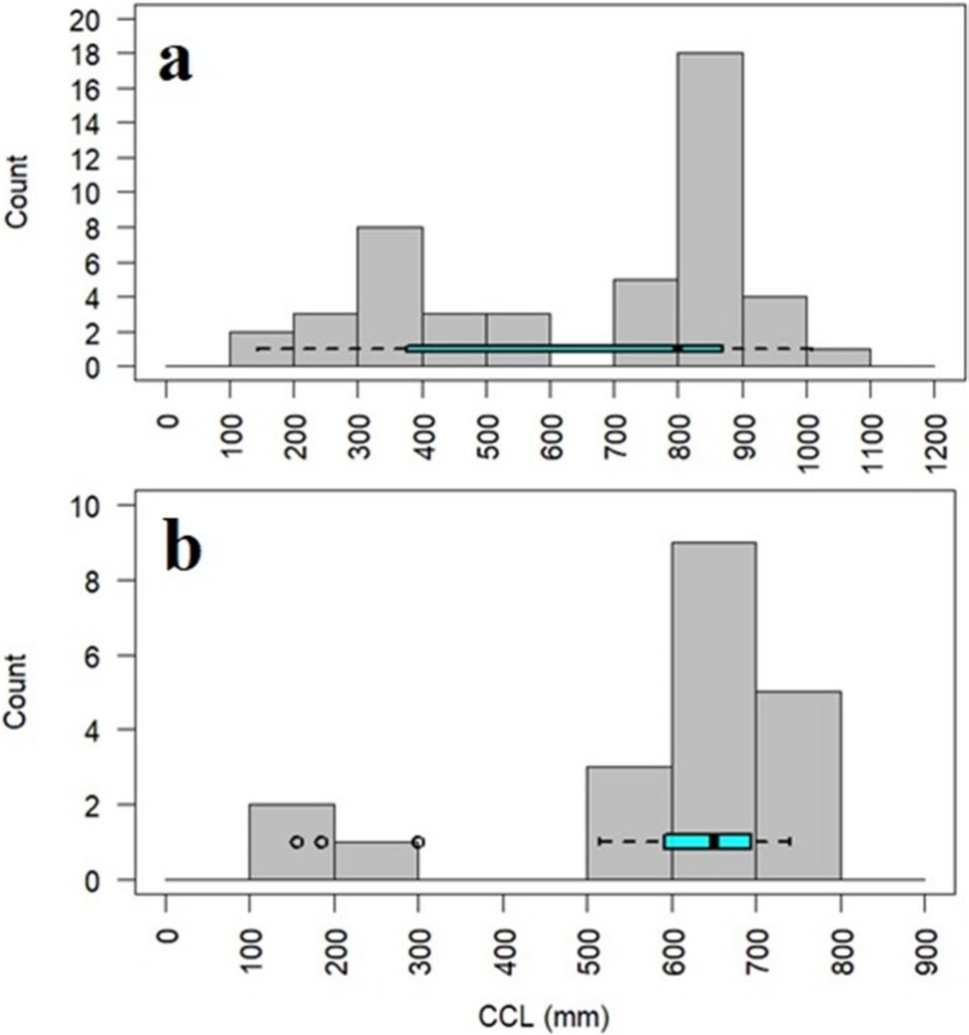
The CCL frequency distribution and box-plot graph of stranded *C. mydas* (**a**) and *C. caretta* (**b**) (1st quartile 377.5, median 800, and 3rd quartile 870 for *C. mydas*, and 1st quartile 596.2, median 650, and 3rd quartile 687.5 for *C. caretta*)

A total of 67 sea turtles (47 *C. mydas* and 20 *C. caretta*) were sampled from Samandağ beach. As Samandağ Beach is the main nesting beach for *C. mydas*, most of the strandings were of this species. The mean CCL of *C. mydas* was measured as 656.6 mm ± 264.5 (145–1010 mm) and as 589.2 mm ± 172.7 (155–740 mm) for *C. caretta*. The CCL frequency distribution and boxplot graph of both stranded sea turtle individuals are shown in Fig. 2.

### Consistency of the number of LAG estimated by readers

**Fig. 3:**
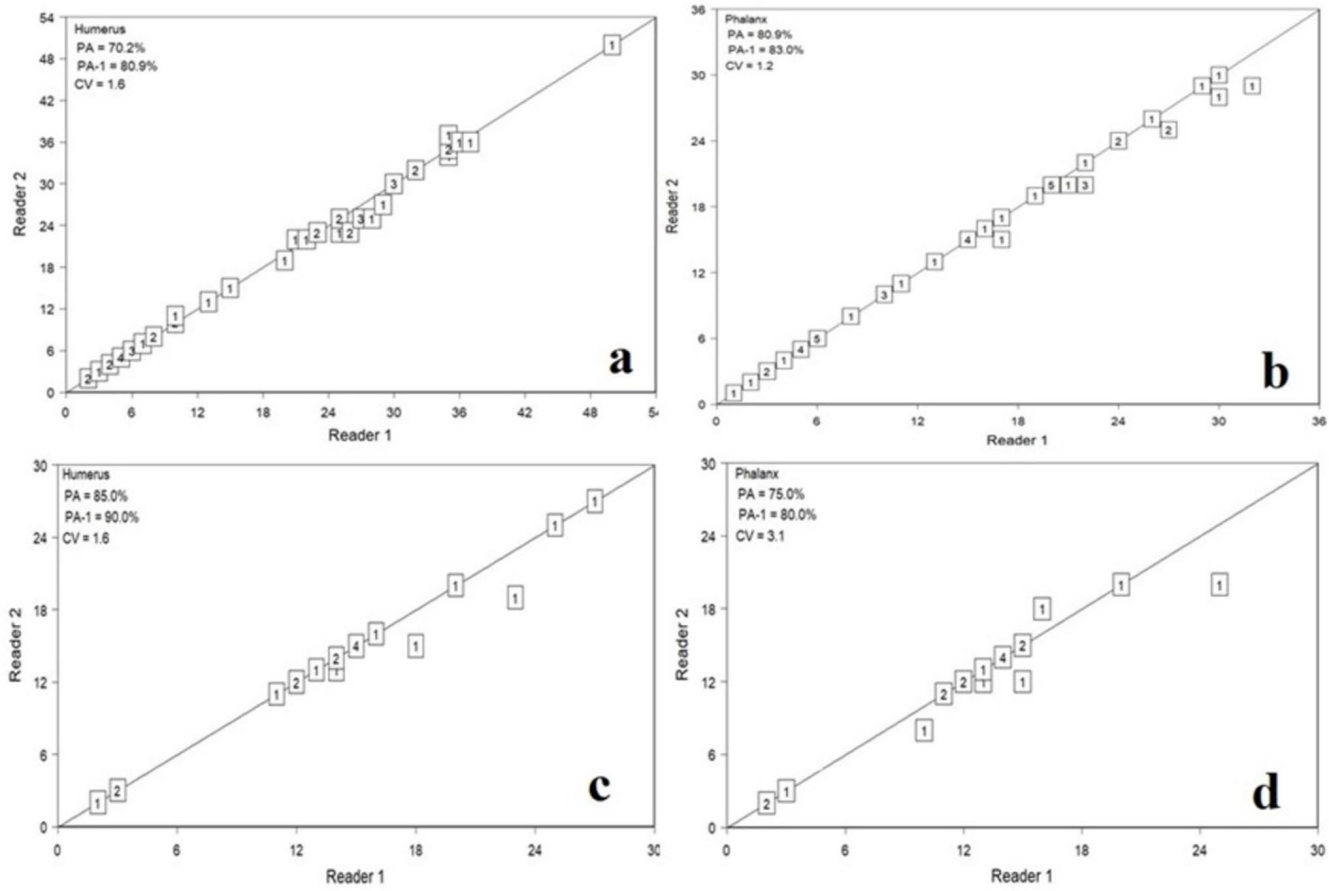
The relationships between the estimated number of LAG in the two readers by species and tissue (**a** humerus and **b** phalanx for *C. mydas* and **c** humerus and **d** phalanx for *C. caretta*. The diagonal line means 1:1 and the graph shows the deviation of the visible LAG count read by the two readers from the 1:1 line. The numbers in the box represent the number of samples at the corresponding LAG count)

A comparison of LAG counted by readers in two different tissues of both species is given in Table 1. There was no significant difference between the visible LAG counted by the two readers in both *C. mydas* and *C. caretta* for humerus and phalanx (*p* > 0.05). The number of LAG estimated by both readers in both bone tissues exhibited high PA and low APE and CV (Table 1). Furthermore, the relationships of the counted number of visible LAG of the two readers by species and tissue are given in Fig. 3.

**Table 1:** Comparison of the LAG count read by the readers in two different tissues of both species (*APE*, average percentage error; *CV*, coefficient of variation; *PA*, percent agreement; *n*, number of specimens; *sd*, standard deviation)

Species	Tissue	Readers	*n*	Mean ± sd	APE ± sd	CV ± sd	PA (%)
*C. mydas*	Humerus	1	47	19.7 ± 12.3	1.1 ± 1.9	1.6 ± 2.7	70
		2		19.3 ± 12.1			
	Phalanx	1		15.2 ± 8.8	0.8 ± 1.8	1.2 ± 2.5	80
		2		14.8 ± 8.4			
*C. caretta*	Humerus	1	20	14.4 ± 6.5	1.1 ± 2.8	1.6 ± 4.1	85
		2		14 ± 6.2			
	Phalanx	1		12.5 ± 5.4	2.2 ± 4	3.1 ± 5.7	75
		2		12.1 ± 5

### Consistency of estimated number of LAG in humerus and phalanx

Since there were no significant differences between the estimated number of LAG by the two readers, the estimated LAG count in each bone represents the average of the two readers. The APE of the estimated mean visible LAG counts in two tissues was 10.6 ± 11.4 for *C. mydas* and 7.26 ± 8.1 for *C. caretta*. Also, the estimated mean visible LAG count in two different tissues was inconsistent with each other, and the PA was calculated as 34% for *C. mydas* and as 40% for *C. caretta*. In the McNemar test, the two different tissues showed significant differences, and it was concluded that the estimated mean visible LAG count was not consistent with each other (McNemar test: *X*^2^ = 13, df = 1, *p* < 0.001 for *C. mydas*, and *X*^2^ = 8, sd = 1, *p* = 0.0046 for *C. caretta*).

**Fig. 4:**
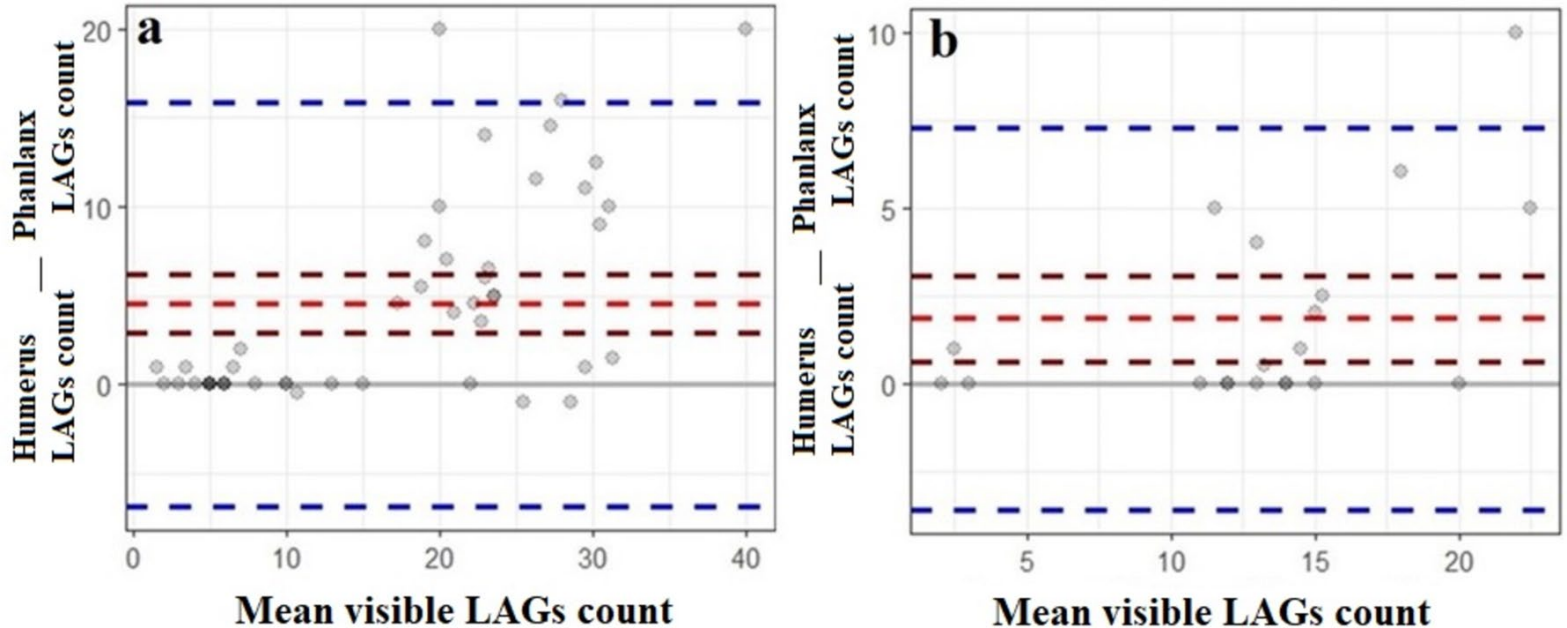
Consistency of LAG count estimated in two different tissues in *C. mydas* (**a**) and *C. caretta* (**b**) (the gray line indicates that the LAG count difference is zero, the red dashed line indicates the mean LAG count difference, the dark red dashed lines indicate the 95% lower–upper confidence interval of the mean LAG count difference, and the blue dashed lines indicate the 95% lower–upper confidence interval of the standard deviation of the LAG count difference)

According to the Bland–Altman plot, the estimated LAG of both tissues in *C. mydas* and *C. caretta* are similar in smaller LAG counts (Fig. 4). In *C. mydas* individuals, LAG estimates are consistent between the two tissues up to a mean visible LAG count of 15; however, there are discrepancies in higher LAG counts (Fig. 4a). Similarly, in *C. caretta* individuals, LAG estimates are consistent for individuals up to a mean visible LAG count of 10. However, discrepancies are observed for higher LAG count (Fig. 4b).

In *C. mydas*, 38 samples of humerus and phalanx tissue from the same individual were selected. The mean resorption rate in the humerus was 1.6 ± 0.35 mm (0.2–2.4) and in the phalanx 1.91 ± 0.50 mm (1.1–3), and a significant result was found between the mean resorption rates (*t* = 2.02, sd = 36, *p* < 0.05). In *C. caretta*, 17 samples of humerus and phalanx tissue from the same individual were selected. The mean resorption rate was 1.64 ± 0.20 mm (1.35–2.19) in the humerus and 1.68 ± 0.26 mm (1.2–2.3) in the phalanx, and there was an insignificant difference between the mean resorption rates (*t* = 2.1, sd = 17, *p* < 0.53).

## Discussion

In this study, the mean LAG count estimated from the humerus was greater than the mean LAG count estimated from the phalanx. Clearly, the humerus contains more growth marks than the phalanx. The humerus has been used in most sea turtle ageing studies across all species as it provides the highest ratio of cortical to cancellous bone (Avens and Goshe 2007; Snover et al. 2007; Şirin and Başkale 2021; Turner Tomaszewicz et al. 2022). Although limited in sea turtles, the use of phalanx in other reptile species has increased in recent years, allowing adequate age estimation without sacrificing individuals (for a review see Szekely et al. 2024 for details). Guarino et al. (2004) reported that cross-sections of the phalanx were well defined and showed grossly concentric LAG. They also performed an osteometric analysis, which calculated the maximum and minimum values of the resorbed LAG. It was reported that the tissue of the *C. caretta* phalanx was markedly altered in the medulla because of remodeling processes and that prominent LAG were observed in all phalanx sections, apart from those of hatchling specimens (Guarino et al. 2020).

Di Maio et al. (2003) documented that the phalanges showed well-defined and concentric LAG in all bone sections and that these were fully consistent with the number and patterns of LAG observed in humerus sections. In contrast, in the present study, the accuracy of the number of LAG estimates in both bones varied with visible LAG count in both species. We confirmed that the humerus and phalanx can be equally useful based on the number of visible LAG, up to 15 for *C. mydas* and up to 10 for *C. caretta*. Guarino et al. (2020) reexamined 13 humerus of *C. caretta* and estimated their age together with the phalanx. They found that the estimated age of the phalanx was in 85% agreement with that of the humerus. However, the high agreement between humerus and phalanx includes individuals between 2 and 15 years of age. Furthermore, Guarino et al. (2004) assumed that one to two LAG were completely resorbed in phalanx for *C. caretta* individuals with a curved carapace length (CCL) between 24 and 38 cm.

It has been stated that it will be difficult to distinguish and count the growth lines in the phalanx bones in later ages (Guarino et al. 2004). Similarly, in this study, significant resorptions were observed in the early growth marks in both bones and the number of LAG observed in the phalanx decreased compared to the humerus. The results demonstrated significant differences between the mean resorption rates in both bones in terms of calculated resorption rates in this study, with a notably higher resorption rate observed in the phalanx of *C. mydas.* The number of LAG observed in 50 individuals exhibiting a CCL length greater than 16 cm in the *C. caretta* phalanx bone in the Mediterranean was reported to range between 3 and 15, while the number of lost LAG was reported to range between 2 and 16 (Guarino et al. 2020). In Atlantic *C. mydas*, the number of LAG with a first age mark has been documented to range from 1 to 17, with the age of the individual equaling the number of LAG within this range (Goshe et al. 2010). It was demonstrated that the estimated number of resorbed LAG in *C. caretta* individuals, as measured using humerus bone, ranged between 0 and 24, and the estimated number of resorbed LAG was 0 for up to 30 cm straight carapace length (SCL), and ≤ 9 for between 30 and 80 cm SCL (Avens et al. 2013). Also, Goshe et al. (2010) found that the first-year annulus was lost to resorption in all green turtles greater than about 45 cm SCL.

The back-calculation and/or correction factor for LAG lost due to resorption play a key and critical role in skeletochronological ageing studies. The statistical methodology behind this is well developed (Snover et al. 2007; Goshe et al. 2010; 2016; Avens et al. 2015; Turner Tomaszewicz et al. 2022; Medetian and Miaud 2024). This statement gives the impression that using different bone types is the solution, but it is not. One of the key findings of this study was that the number of LAG observed differed between the phalanx and humerus. This was supported by resorption rates, indicating that the number of LAG lost due to resorption may also differ between these two bone types. It is therefore recommended that the back-calculation and/or correction factor applied for the humerus should be avoided for the phalanx, although the use of the phalanx shortened the preparation time of decalcified sections and offered advantages in the preparation of tissue sections (Guarino et al. 2004). Furthermore, the relationship between the equation defined as the resorption rate and the number of LAG contained in the bones can be subjected to further analysis in future studies. Thus, correction equations for absorbed LAG can be developed.

One of the important limitations of this study is that it was not carried out on specimens of known age. Therefore, it would be inaccurate to conclude the accuracy of age estimates in both bones without samples of known age. Therefore, the important findings of this study are the consistency of visible LAG count between bone types and the results of LAG lost to resorption. A further significant limitation is the absence of specimens representing all carapace lengths for both species. Notably, the limited number of juveniles (i.e., individuals < 50 cm) for *C. caretta* does not allow for adequate comparison with adults, which may introduce bias. In subsequent studies, it is necessary to include all carapace lengths of individuals to mitigate this bias.

In conclusion, the humerus contained more LAG count than the phalanx for both species. The phalanx exhibited high resorption of early growth marks and up to a certain number of LAG (15 for *C. mydas* and 10 for *C. caretta*), both bony structures can be used as a reliable structure for age determinants. Although age-related information is necessary for the management and conservation planning of these two species, there are few studies on age estimation by phalanx in *C. caretta* and no studies (both humerus and phalanx) on *C. mydas* in the Mediterranean. Therefore, in studies to be carried out in the Mediterranean for both species, there is a need to confirm the ages determined in two different bone tissues with samples of known age.

## Data Availability

Data will be made available on request.
